# Itga5-PTEN signaling regulates striatal synaptic strength and motor coordination in Parkinson's disease

**DOI:** 10.7150/ijbs.96116

**Published:** 2024-06-11

**Authors:** Bei Zhang, Yong-Bo Hu, Gen Li, Hong-Xiang Yu, Can Cui, Ying-Ying Han, Hong-Xia Li, Gang Li

**Affiliations:** 1 Department of Neurology, Shanghai East Hospital, School of Medicine, Tongji University, Shanghai 200092, China.; 2 Department of Neurology, Huashan Hospital, Fudan University, Shanghai 20040, China.

**Keywords:** Parkinson's Disease, Itga5, PTEN, Synaptic Integrity, Motor Coordination, MPTP Model, Therapeutic Target

## Abstract

**Background**: Parkinson's disease (PD) is marked by the loss of dopaminergic neurons in the substantia nigra pars compacta, leading to motor and cognitive dysfunctions. The molecular mechanisms underlying synaptic alterations in PD remain elusive, with a focus on the role of Itga5 in synaptic integrity and motor coordination and TAT-Itga5 was designed to suppress PTEN activity in this investigation.

**Methods**: This study utilized MPTP-induced PD animal models to investigate the expression and role of Itga5 in the striatum. Techniques included quantitative PCR, Western blotting, immunostaining, CRISPR-CasRx-mediated knockdown, electrophysiological assays, behavioral tests, and mass spectrometry.

**Results**: Itga5 expression was significantly reduced in MPTP-induced PD models. In these models, a marked decrease in dendritic spine density and a shift towards thinner spines in striatal GABA neurons were observed, suggesting impaired synaptic integration. Knockdown of Itga5 resulted in reduced dendritic branching, decreased mushroom spines, and increased thin spines, altering synaptic architecture. Electrophysiological analyses revealed changes in action potential and spontaneous excitatory postsynaptic currents, indicating altered synaptic transmission. Motor behavior assessments showed that Itga5 deficiency led to impairments in fine motor control and coordination. Furthermore, Itga5 was found to interact with PTEN, affecting AKT signaling crucial for synaptic development and motor coordination.

**Conclusion**: The study demonstrates that Itga5 plays a critical role in maintaining synaptic integrity and motor coordination in PD. The Itga5-PTEN-AKT pathway represents a potential therapeutic target for addressing synaptic and motor dysfunctions in PD.

## Background

Parkinson's disease (PD) is a degenerative disease of the nervous system that causes problems with movement, such as tremors at rest, stiff muscles, and walking difficulties 1, 2. Pathogenesis is characterised by piecemeal deterioration of dopaminergic neurons in the substantia nigra pars compacta and decreasing dopamine levels in the putamen of the dorsolateral striatum 3. PD occurs in the loss of dopaminergic afferents to the striatum as well as striatal output via the direct and indirect pathways, which are two parallel basal ganglia circuits crucial for motor function and procedural learning. Mobility is restricted because of these changes 4-6. The striatum serves as the convergence site for glutamatergic inputs originating from the cortex and cerebral ganglia, as well as dopaminergic afferents originating from the mesencephalon. Understanding the physiology of the striatum is crucial for appreciating the symptoms associated with Parkinson's disease 7-9.

The main neuronal subtypes are medium spiny neurons, which form the bulk of striatal neurons 10, 11. Medium-sized spiny neurons (MSNs) accept glutamatergic imports from the cortex and cerebral ganglion, which end on lumbar vertebrae 12. Dopaminergic neuron axons in the midbrain also connect to MSN dendrites and spine necks 9. In PD, striatal dopamine denervation lowers the number of dendritic spines on medium spiny projection neurons. Striatal synapses undergo ultrastructural remodeling in answer to variation in synaptic activity, leading to complex morphological, neurochemical, and electrophysiological striatal synaptic transmission anomalies 13-16. Synaptic remodeling is a fundamental mechanism for motor regulation and learning in the striatum. However, the specific processes driving striatal MSN synaptic changes and motor impairments in unwell persons remain unclear.

The investigation of genetic or molecular anomalies in PD has been facilitated via the use of single nucleotide polymorphisms and genome-wide association studies 17. After investigating gene expression levels in the substantia nigra (SN), it was discovered that a considerable number of genes engaged in synaptic function and cytoskeletal conservation are downregulated in the human parkinsonian nigrostriatal pathway. In the striatum, many genes in the cell adhesive family are turned down, and synaptic genes are likewise downregulated 18. Genome-wide association researchers have found a high amount of unitary nucleotide pleomorphisms within cell adhesion pathways 19. Cell adhesion is related with three of the top five pathways most likely to be disturbed in PD, including focal adhesions, adherens junctions and cell adhesion molecules, showing that cell adhesion failure may play a role in disease start 20. Cell adhesion molecules connect the pre- and post-synaptic areas and help with trans-synaptic recognition and signaling. These are important for the formation, specification, and plasticity of synapses, which allows neurotransmission. Adhesion difficulties can lead to structural alterations at synapses and other cell regions, which can contribute to neurodegeneration 21-23. Despite these findings, there have been little investigations on cell adhesion issues in PD.

In order to investigate this phenomenon, researchers conducted a study on cell adhesion molecules that exhibit high expression in striatal neurons innervated by nigral dopamine (DA) in mice treated with MPTP. The findings of this investigation revealed a significant drop in the levels of integrin a5 protein (Itga5). Using CAS-RX-specific knockdown, we found that Itga5 is necessary for synaptic integrity and development, as well as motor activity. PTEN is a newly identified protein that regulates AKT signaling by interacting with Itga5. To restore motor function, Itga5 C-terminal binding peptide is injected into striatal neurons of MPTP-treated rats. As a result, our findings lead to a novel synaptic adhesion signaling regulation mechanism as a possible therapy target for PD.

## Materials and Methods

### Animals

Institutional and government rules were followed while taking care of animals. All the mice were kept in a room that was 22-25°C and had food and water available 24 hours a day. Male C57BL/6 mice 8 to 15 weeks old and Vgat-ires-cre knock-in mice were used in this study.

### Behavioral evaluation

Mice aged 8 to 12 weeks were subjected to behavioral tests. During the transition between the light and dark phases (18:00-20:00), all behavioral tasks were carried out. Mice were put in the room three hours before the activities started to get them used to the test setting. The PD mouse model was constructed using a previously reported approach 24. For five days, mice were supplied with 30 mg/kg, 1-methyl- 4-phenyl-1, 2, 3, 6-tetrahydropyridine (MPTP) dissolved in 0.9 percent saline intraperitoneally (IP), where the control group got the same amount of saline (IP). The open field test, footprint test, and rotarod test were performed to assess behavior two days after the last inoculability.

### Open field test

Mice were placed in the middle of an open field made of Plexiglas that was 40 cm by 40 cm by 35 cm. They were then given 30 minutes to explore. To figure out the total distance and time spent in the center, EthoVision video tracking equipment was used (Noldus Information equipment).

### Footprint test

The footprint test was done exactly as detailed in the preceding section 25. The mouse's paws were sprayed with non-toxic paint before the rodent was permitted to stroll down a short, paper-covered hallway, creating a painstakingly recorded footprint trail. The stride length was estimated by averaging the distance between strides. The distance between the left and right front or rear footprints can hele us obtain the width of the base. The registration between forepaw and hind paw positioning was explored by measuring the interval between the front and rear footprints on the same side, which was then employed to determine accurate foot placement and step alternation consistency.

### Rotarod test

To measure motor performance, a commercially available rotarod (Ugo Basile) was employed. Accelerating and progressive fixed-speed approaches were applied. To alleviate stress and exhaustion, two trials were conducted to accelerate the mouse to 40 rpm in 5, 3, or 1 minute, separated by a 30-minute rest period. The mouse was placed on a rotarod with a maximum speed of 40 rpm and tested for five minutes. We examined the mice three times each day, with a 30-minute delay between each observation. The mouse's time spent sliding down the moving rod was recorded.

### Beam walking test

The beam walking test was done exactly as stated in the prior section 25. Finally, one-meter-long wooden beams were utilized. Over the course of two days, mice were taught to transverse a square beam with a moderate width 12 mm (three trials per day). The majority of mice were able to thread the 12-mm beam in 20 seconds following training. The coached mice were tested on six varied types of beams two days later, beginning with round 28 mm diameter beams and square 28 mm width beams. On each beam, two 10-second interval test attempts were done, and the average time the mouse required to traverse the 80-centimeter center area was recorded and examined. A time constraint of 60 seconds was set. Because the majority of mice were unable to walk across the 5-mm square beam, their motor competence was measured by measuring their walking distance on it.

### Real-time PCR

The TRIzol procedure was used to extract total RNA from dissected striatal tissue (Invitrogen). Four micrograms of overall RNA were employed as a template for supplementary DNA (cDNA) synthesis and enhancement utilizing high-capacity cDNA reverse transcription kits, as indicated by the manufacturer (Applied Bio systems). Following PCR amplification, 100 mg of diluted cDNA was employed. The Takara SYBR Premix Ex Taq kit was used with the applied Bio systems ABI PRISM 7000 Sequence Detection System under the next enhancement circumstances, 95°C for 5 minutes, 40 cycles of 95°C for 15 seconds, 60°C for 15 seconds, and 72°C for 31 seconds. Table 1 shows the primers for real time PCR.

### Western blot analysis

Brain tissue was attenuated 20-fold (μL/mg) with ice-cold RIPA buffer and sonicated on ice. Following thirty minutes of analysis on ice, the tissue was centrifuged (12,000g, 4°C) at four degrees Celsius for thirty minutes. Using a BCA kit, the protein content of the supernatants was evaluated after their collection. Before immune precipitation, tissues or cells were analysed for one hour at 4°C in lysis buffer. The protein was denatured after 10 minutes of boiling in the loading buffer. On 15% SDS-PAGE gels, identical quantities of protein samples were separated and transferred to PVDF membranes (Millipore, USA). Primary antibodies against actin (1:5000, Thermo Fisher Scientific, ma5-15739), Itga5 (1:500, Millipore, AB1928), Flag (1:1000, Sigma-Aldrich, F1804), HA, and tubulin were applied to these membranes (1:5000, Sigma-Aldrich, T8328). The membranes were incubated for two hours with a minor antibody gemeled to horse radish peroxidase following three 10-minute washes (1:5000, KPL, Gaithersburg, MD, USA). ECL and detection apparatus were utilized to identify protein bands. Using image analysis techniques, the intensity of every protein band was subsequently measured (Quantity One, Japan).

### Immunohistochemistry

Method of immunohistochemistry previously reported 26. The following steps were applied to the STR slices. The STR was extracted from 20 m coronal slices using a cryostat (Leica CM3050S). After being rinsed with 0.1m PBS, pH 7.4, the slides were hatched during the night at 4°C in blocking buffer containing primary antibodies. After three washes in 0.1m PBS, pH 7.4, the sections were incubated at room temperature for 24 hours with a secondary antibody, donkey anti-rabbit IgG H&L. The nucleus has been stained with DAPI. Before covering with coverslips, glycerin was applied to the parts. Utilizing a Zeiss confocal microscope, fluorescent pictures were captured (LSM 880). We used the following major antibodies, rabbit anti-Itga5 (1:200; Millipore, AB1928), rabbit anti-tyrosine hydroxylase, mouse anti-GAD1, and rabbit anti-HA (1:200; Millipore, AB1928) (1:1000, Cell Signaling, 3724).

### Mass spectrometry technique

After three washes in 500 L of 100 mm NH_4_HCO_3_, the beads were sonicated for 30 minutes in 40 L of 8 m urea dissolved in 100 mm Tris-Cl at pH 8.5. The solution was hatched at room temperature for 30 minutes with TCEP (Thermo Scientific) (10 mm final concentration) and iodoacetamide (15 mm final concentration) for reduction and alkylation (Sigma). Prior to being treated with Trypsin at a ratio of 1:50 (w/w), the protein dissolution was diluted fourfold (Promega). After formic acid (FA) was used to stop the digestion, the peptide compound was desalted using a monospin C18 column.

The peptide compound was injected at a flow rate of 300 mL/min onto an Easy-nLC1200 system with an upside-down stage C18 column equipped with a 120-minute buffer B gradient. Before being delivered to an Orbit Rap Q Executive mass spectrometer, the eluted peptides were ionized with a noy-spray provenience and a terminal 2.2-kV spray electrical pressure. With a resolution of 70000 and an AGC goal of 1e6, the MS range was set at 300-1800 m/z. The tandem MSMS was acquired in HCD mode with a maximum injection duration of 100 ms, a resolution of 17500, an NCE of 28% and an AGC target of 1e5. The isolation window was set to 1.8 m/z with a 15 second dynamic exclusion duration.

Using Protein Discoverer 2.1, the obtained MS/MS data were compared to the UniProtKB mouse database. Cysteine alkylation by iodoacetamide was characterized as a fixed change, while methionine oxidation was categorized as a dynamic change. Full specificity mode has been engaged on the trypsin enzyme. Three missing cleavages were the most frequent. To evaluate peptide chances and improper detection rates, we created an inveiglement database including the adverse order of the whole proteins linked to the aimed database. The FDR for peptide-spectrum matching was 1 percent. The mass error tolerance for parents was 20 ppm, whereas the mass error tolerance for fragments was 0.02 Da.

### CasRx-containing plasmids

HuaGen produced the CasRx and sgRNA backbone sequences. The CBH-DIO-CasRx-U6-DRs-BbsI-sgRNA vectors were subsequently constructed. BbsI restriction enzyme was applied in order to include sgRNA. Cas13d predicts guide RNA for target transcripts based on the guide RNA design strategies described in the studies listed below. Numerous Cas13 screens disclose the principles of guide RNA design 27. PTEN sgRNA: 5'-CATCTTGAAACAGCAGTGCCA-3'; CasRx sgRNA: 5'-CTACCTGGTAGCCCTTGTATTTG-3'; Itga5 sgRNA: 5'-GGACACACATAGACAGCACCACC-3'.

### Sliced brain electrophysiology

Adult male Vgat-ires-cre mice received an AAV2/9-EF1-DIO-m injection (6 to 8 weeks old). Cherry were anesthetized with 100 mgkg-1 of intravenous pentobarbital and transcardially perfused with a 95/5 oxygenated NMDG ACSF dissolution. This solution consisted of 93 mm NMDG, 93 mm HCl, 2.5 mm KCl, 1.25 mm NaH_2_PO_4_, and 10 mm MgSO_4_7H_2_O. After perfusion, the brain was fast shifted and put in an icy NMDG ACSF solution including oxygen. At 300 m in the same buffer, the coronal plane of brain tissue was segmented utilizing a vibratome. VTA and NAc were hatched in oxidative NMDG ACSF at 32°C for 10 to 15 minutes before being shifted to a regular oxidative ACSF dissolution.

The portions were then moved to the recording room, which was underwater in ACSF and subcooled at a ratio of 3 mL min-1 at a temperature of 28 degrees Celsius. Dopamine-active neurons were discovered using differential interference contrast optics. Utilizing a micropipette puller, three to four millimeter recording pipettes were created (P2000, Sutter Instrument USA). A CSF was used for whole-cell recordings (pH: 7.2, 280 mOsm). For action possibilities elicited by present injections (from 25 to +300 pA, with a 25 pA increment between each STR recording), a current-step technique was developed and repeated. Voltage-clamp mode was applied to the neurons for 5 minutes at -70 mV to capture spontaneous excitatory postsynaptic currents (sEPSCs). The statistical analysis eliminated records with R values over 30 million. All signals were 3 kHz low-pass filtered and 10 kHz digitalized with a Multiclamp 700B microphone. Utilizing Clamp fit 10 and the Molecular Devices Mini Analysis Program, the physiological data were analyzed (Synapto soft).

### Viral injection and cannula infusion

Injection of a virus and implantation of a cannula have previously been reported 28. We adopted Bregma and Lambda points to regulate the horizontal location of the mouse's head. At the site of viral jabs and cannulae transplantation, a fester with a diameter of 300 to 500 microns was created. The injection ratio of the virus was 300 ml/min. This study utilized AAV2/9-DIO-GFP, AAV2/9-DIO-CasRX, AAV2/9-DIO-CasRX-Itga5 and AAV2/9-DIO-CasRX-PTEN. The device was posed at least 10 minutes after injection. After 14 to 20 days of recovery, the mice were utilized for further research. For the cannula infusion experiment, the double guide cannula (2.2 mm center-to-center distance, RWD) was positioned above the mice's NAc. Placement of the cannula above the NAc; 1.18 mm AP; 1.3 mm ML; 2.0 mm DV to prevent clogging during the recovery phase, the guiding cannula was equipped with a double-dummy cannula (RWD) and a dust cap. After at least 10 days of recovery, the Itga5 peptide was microinjected using a double injector cannula that lengthened 0.5 mm overtop the tip of the guide cannula. The double injector cannula was placed into and then extracted from the guide cannula to guarantee a clean path prior to the local medication infusion. Using a micro syringe-connected double injector cannula (0.1 l/min), 1 l (2 M) of peptides were injected into both sides. The injector cannula was maintained in a position for an extra five minutes, to prevent the Itga5 peptide from spreading along the injection line. On the tenth day, following five days of daily AAV virus injections, 0.1 l/min of Itga5 peptide was injected into each side of mice (double injector cannula) to downregulate Itga5. For the Itga5 peptide in MPTP treatment experiment, mice were injected with (0.1 l/min) Itga5 peptide by double injector cannula into each side at the beginning of 5 days of daily MPTP administration.

### Sholl evaluation and spine evaluation

The Simple Neurite Tracer plugin for Fiji/Image J was utilized to reconstruct dendritic branches on MSN neuron images (NIH, Bethesda, MD, USA). Using Image J's Sholl analysis, the number of dendritic branches was determined. Rodriguez et al. 29 categorized the morphology of neuronal backbones in slices utilizing Neuron Studio software and an algorithm with the key values aspect ratio. Spines with HNR more than HNR (crit) can be thin or mushroom, backbones with HD greater than HD (crit) are mushroom, or else thin and backbones without chief necks and less than AR thin (crit) are stubby, or else thin. The spine density was computed by multiplying the total number of spines by the length of the dendritic branch. Filo podia, defined as protrusions longer than five meters, were eliminated from the study.

### Using electron microscopy

For 30 minutes, mice were given 2% PFA/2.5% glutaraldehyde in buffer of phosphate, pH 7.2. The brains were taking out, post fixed in 2% PFA/2.5% glutaraldehyde overnight, washed in PBS, and vibratome-sliced into 200 m slices. After being post fixed with 2% osmium, the pieces were dried and encased in Araldite. On a CM-120 machine, ultrathin slices (70 nm) were cut, colored with lead citrate, and photographed at 17500 x magnification (Philips). In the STR region, we took an average of 10 photographs per mouse (n = 3 per group). Image J was utilized while blinded to determine the thickness and length of striatal PSD.

### Statistical analysis

Graph Pad Prism 8 was used for all statistical analysis. Depending on the conditions, one-way ANOVA and two-way ANOVA both followed by the test of Dunnett, the paired or unpaired t-test, or the paired or unpaired t-test were applied. When the variance of the data has substantial differences, the test of Kruskal-Wallis was applied instead of Tukey's test. Using a p-value of 0.05, group differences were deemed significant statistically. Asterisks express statistical significance: *p 0.05; **p 0.01; ***p 0.001; ****p 0.0001. These numbers represent the mean standard deviation.

## Results

### Itga5 expression was lowered in PD animal models induced by MPTP

**Figure 1:**
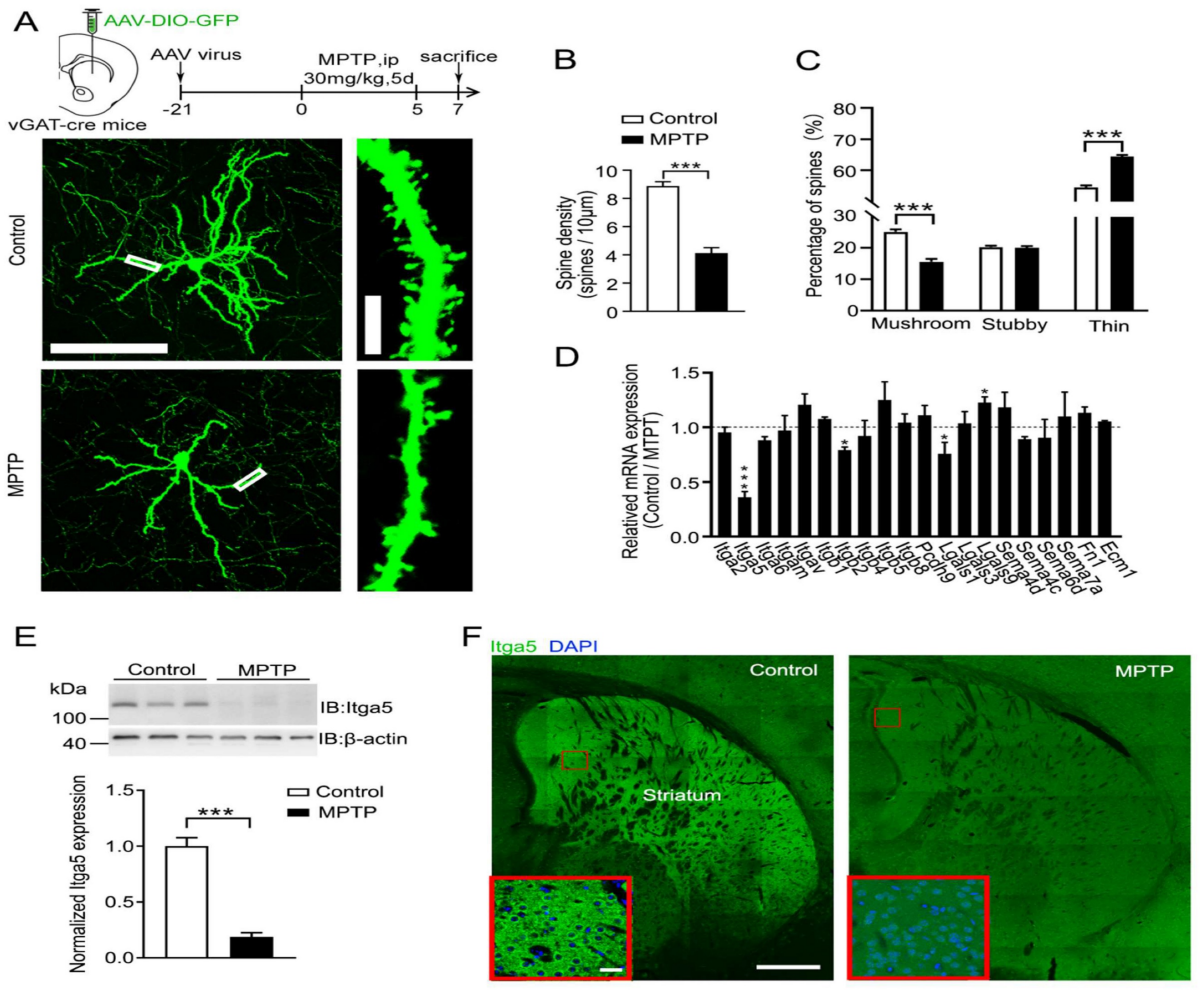
(A) AAV-DIO-GFP was stereo tactically placed into the STR of vGAT-cre mice. (B) The number of spines per dendritic length was less in MPTP-induced PD model mice than in control mice (C) MPTP-induced PD model animals exhibited considerably more thin spines and fewer mushroom spines than control mice. (D) Gene expression in dissociated striatal tissue treated with MPTP to uncover putative cell adhesion molecules related with PD. (E and F) Quantitative PCR and western blotting and immunostaining.

The striatum (STR) is the major nucleus group projected by the substantia nigra neurons, and its necrosis is the hallmark of PD. The morphology of STR neurons in MPTP-induced PD animal models was studied to see if it has altered. AAV-DIO-GFP was stereo tactically placed into the STR of vGAT-cre mice to identify STR GABA neurons (Fig.1A). GABA neuron postsynaptic integration was found utilizing the dendritic spines of GFP-labeled GABA neurons in the STR. The number of spines per dendritic length was considerably less in MPTP-induced PD model mice than in control mice (Fig. 1A-1B), showing acute synaptic loss of STR GABA neurons in MPTP-induced PD model animals. MPTP-induced PD model animals exhibited considerably more thin spines and fewer mushroom spines than control mice (Fig. 1C), indicating that striatal neuron postsynaptic integration in these mice was impaired. After infusing depolarizing current through a recording electrode positioned on the soma of STR GABA neurons. We found a considerable increase of value for action potentials (AP) in MPTP-induced PD model animals (Fig.1A-1B). However, in MPTP-induced PD model animals, both the frequency and amplitude of spontaneous excitatory postsynaptic currents (sEPSC) were lower than in normal mice shown in Figure 1C-1D. According to these findings, MPTP affects the synaptic architecture and function of STR GABA neurons, which is consistent with PD.

**Figure 2:**
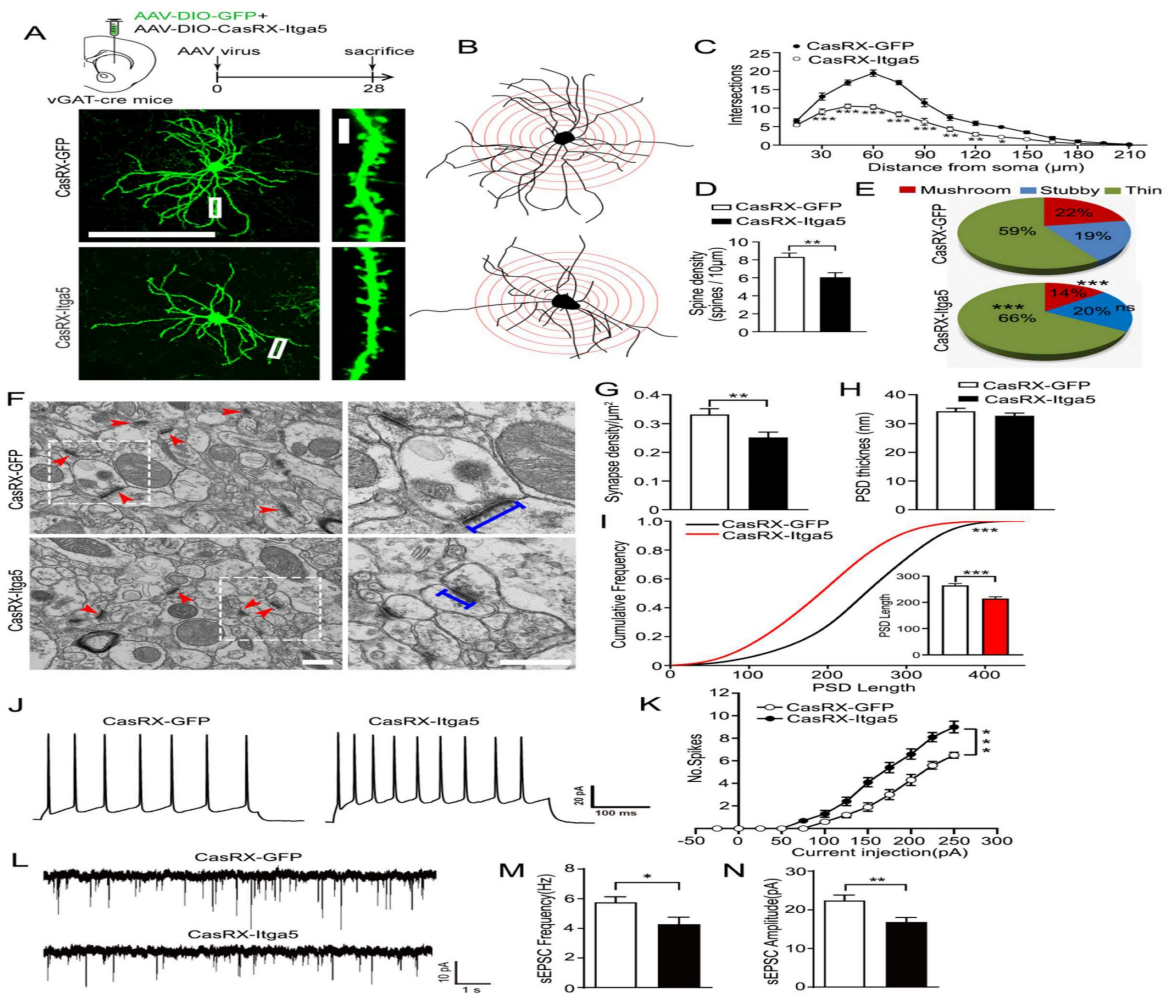
(A, B, C) Inhibiting the Itga5 protein reduced the number of dendritic branches on GABA neurons relative to a GFP control. (D) In the Itga5 knockdown group, the number of thin spines increased while the density of dendritic spine and the ratio of mushroom spines decreased considerably. (E) The inhibition of Itga5 led to a decrease in mushroom spines and an increase in thin spines, prompting more research into synaptic architecture. (F, G, H) Using electron microscopy, we evaluated the size of PSD, an electron-dense area found in the head of spine in the striatum. (I) The CasRX-Itga5 group had a drop in synapse density and PSD lengths. (J, K) In CasRX-Itga5 mice, the number of action potentials (AP) increased but the amplitude and frequency of sEPSC decreased. (L, M, N) Expression of Itga5 is diminished in the striatum.

Then we investigated for substances that were numerous in striatal GABA neurons that may be causing MPTP-induced poor postsynaptic integration. We focused on cell adhesion molecules for several reasons. Dopaminergic neurons in the SNc project to the STR, which needs cell adhesion molecules to create the nigrostriatal projection. Cell adhesion molecules influence pre- and post-synaptic integration and transmission. We looked examined gene expression in dissociated striatal tissue treated with MPTP to uncover putative cell adhesion molecules related with PD shown in Figure 1D. The expression of the integrin 5 (Itga5) gene was considerably reduced in MPTP-induced PD model mice compared to control mice, as measured by quantitative PCR and verified by western blotting and immunostaining (Fig.1E-1F). Our results confirm that Itga5 is highly expressed in the STR region, as reported by the Allen brain atlas database displayed in Figure 2A-2C. Itga5 co-localizes with the GABA neuron marker GAD1, showing that it is only present in striatal GABA neurons. Itga5 may be implicated in the pathogenic mechanism of PD based on our findings and the fact that Itga5 expression was decreased in the peripheral blood of early PD patients expressed in Figure 2 30.

### Itga5 loss inhibits striatal GABA neuron synaptic function

**Figure 3:**
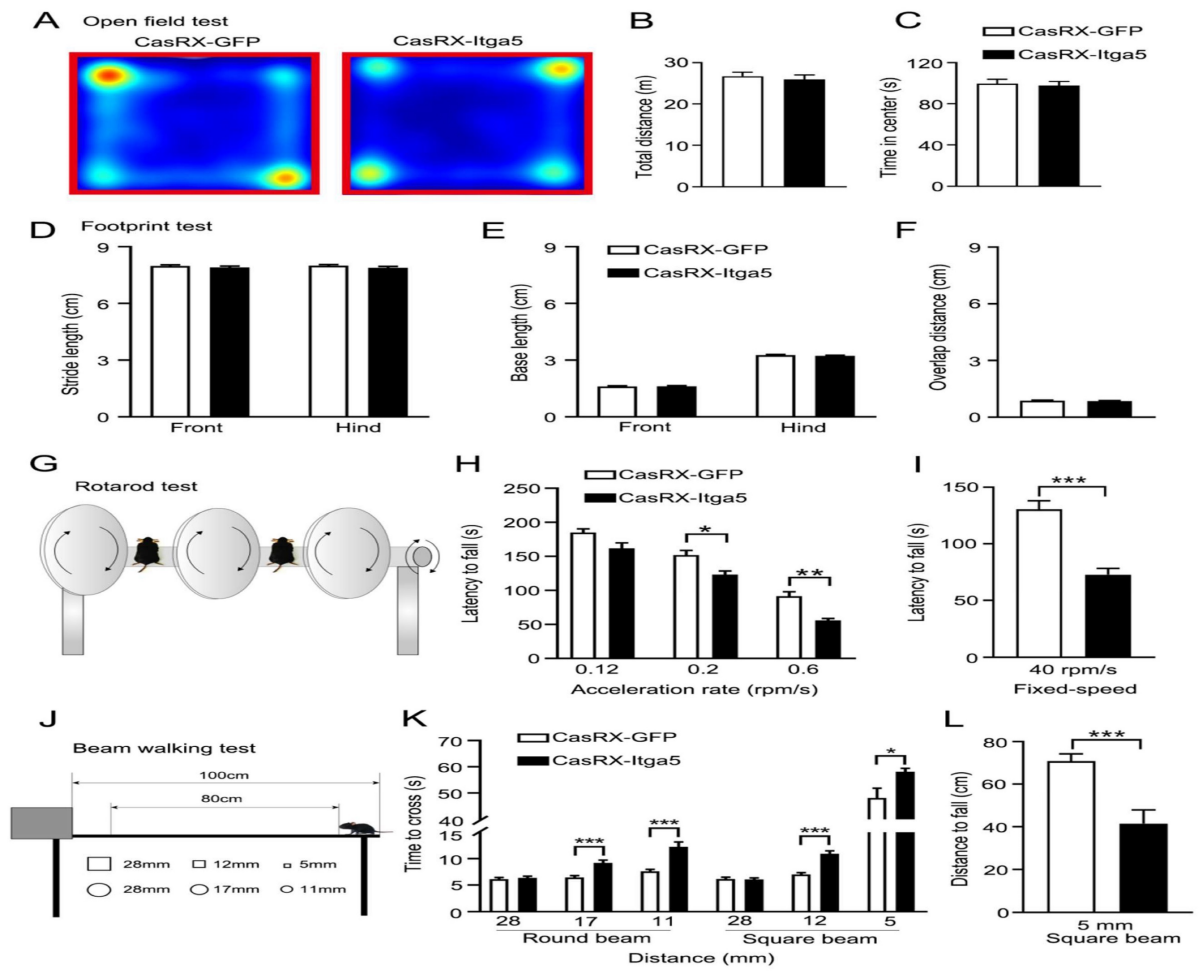
(A, B, C) distances were traversed without any symptoms of worry. (D) Front and back stride lengths of mice in the GFP and CasRX-Itga5 groups were comparable. (E) The left and right front and rear paws have identical base widths. (F) The overlapping placement between forepaw and hind paw did not change between the GFP and Itga5 groups. (G, H, I) CasRX-Itga5 mice performed much poorly and fell more sooner when acceleration rates were increased. (J, K, L) The CasRX-Itga5 mice required much more time to traverse the horizontal beams of varying dimensions and shapes during the balance beam test.

To determine if the structure and behavior of PD-like neurons changed, we knocked down the Itga5 protein in striatal GABA neurons using the CasRx system. CasRx, the smallest member of the Cas13 protein family has knockdown effectiveness equivalent to or greater than RNAi, but with far fewer off-target effects, making it potentially more beneficial for therapeutic applications 31-33. To inhibit Itga5 protein in striatal GABA neurons, an AAV virus expressing DIO-Cas-RX-Itga5 was injected into the STR of 3-month-old vGAT-cre mice shown in Figure 3A-3B. Figure 3C-3F shows that STR Itga5 knockout has no effect on SNc or nigrostriatal projections. The researchers next examined how diminished Itga5 activity affects the number and shape of dendritic spines on striatal MSNs. Sholl discovered that inhibiting the Itga5 protein reduced the number of dendritic branches on GABA neurons relative to a GFP control (Fig. 2A-2C). In the Itga5 knockdown group, the number of thin spines increased while the density of dendritic spine and the ratio of mushroom spines decreased considerably expressed in Figure 2D-2E. The thin spines are believed to be juvenile excitatory synapses, whereas the mushroom-shaped spines are adult excitatory synapses. The inhibition of Itga5 led to a decrease in mushroom spines and an increase in thin spines, prompting more research into synaptic architecture. Using electron microscopy, we evaluated the size of PSD, an electron-dense area found in the head of spine in the striatum. The CasRX-Itga5 group had a drop in synapse density and PSD length, but not PSD thickness. Figure 2F-I indicating a contraction of the postsynaptic architecture.

By injecting depolarizing current steps into the soma of STR GABA neurons, action potentials (AP) were monitored to see if changes in synaptic density and maturation affect intrinsic excitability and synaptic transmission. When CasRX-Itga5 mice were compared to GFP control mice, the number of action potentials increased but the amplitude and frequency of sEPSC decreased showed in Figure 2J-2K. Our results imply that Itga5 controls the maturation of postsynaptic structures for functional synaptic transmission, which may be the source of MPTP-induced synaptic dysfunction, corresponding with the phenotype observed in MPTP-induced mice models of PD.

Due to the consequence of the striatum for motor control and coordination, various motor-related behavioral studies were conducted to determine the role of Itga5 in motor activities. Injections of AAV expressing CasRx-GFP or DIO-Cas-RX-Itga5 were made in the STR of vGAT-cre mice (CasRX-Itga5). Three weeks later, we compared the baseline locomotor activity of the GFP and CasRX-Itga5 groups in an open field test and found no differences. In 30 minutes, the same distances were traversed without any symptoms of worry shown in Figure 3A-3C. Additional examination of the footprints revealed the locomotor gait. Front and back stride lengths of mice in the GFP and CasRX-Itga5 groups were comparable, as measured by the average distance traveled between each step in Figure 3D. Figure 3E shows the left and right front and rear paws have identical base widths. Furthermore, the overlapping placement between forepaw and hind paw, as determined by the length between each side's front and rear footprints, did not change between the GFP and Itga5 groups (Fig. 3F). The results show that inhibiting Itga5 generates little influences on the locomotion and locomotor gait of mice.

We utilized an accelerated rotarod and a balancing beam to determine if mice lacking Itga5 have poor fine motor control or coordination. At moderate acceleration speeds (0.12 rpm/s), no significant variation was found between the GFP and CasRX-Itga5 groups in terms of their ability to maintain balance or fall latency. CasRX-Itga5 mice performed much poorly and fell more sooner when acceleration rates were increased indicated in Figure 3G-3I. The CasRX-Itga5 mice required much more time to traverse the horizontal beams of varying dimensions and shapes during the balance beam test (Fig. 3J-3L), indicating that Itga5 deficiency impairs the function of coordination maintaining during a motor task. These findings suggest that inhibiting Itga5 in striatal GABA neurons may result in motor impairments comparable to those seen in PD.

### Together, Itga5 and PTEN control AKT signaling

**Figure 4:**
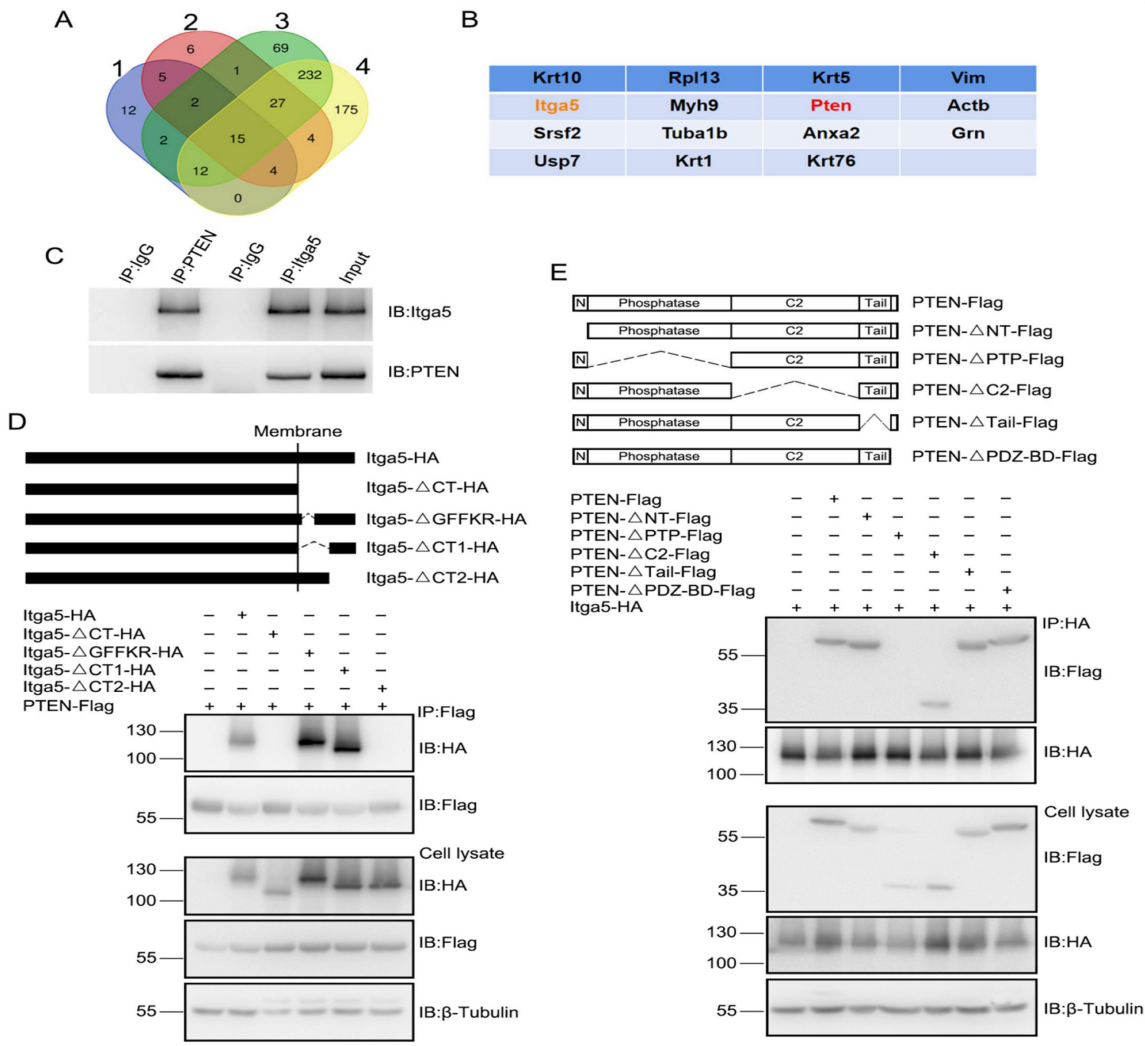
(A, B) The proteins brought down by Itga5 were analyzed by mass spectrometry, and the same 14 proteins were identified in four separate experiments. (C) The PTEN antibody lowered Itga5, and vice versa. (D) Co-transfection of Itga5 HA-tagged domain mutation and Flag-PTEN plasmids, immunoprecipitation for Itga5 or PTEN binding sites. (E) The connection requires the PTEN phosphatase domain, as found by using Itga5 to evaluate probable PTEN binding sites.

Immunoprecipitation (IP) was performed on adult striatal protein lysates using an Itga5 antibody, to investigate the likely molecular mechanism of Itga5 for synaptic development and motor coordination. The proteins brought down by Itga5 were analyzed by mass spectrometry, and the same 14 proteins were identified in four separate experiments shows in Figure 4A-4B. In the central nervous system, PTEN (phosphatase and tensin homolog on chromosome 10) is a protein/lipid phosphatase, that dephosphorylates PIP3 and inhibits the AKT/mTOR pathway for synaptic plasticity and intrinsic excitability 34, 35, 36. After PTEN knockdown in the STR area, we observed an increase in spine density, thin spines, sEPSC frequency, and amplitude, indicating that PTEN plays a part in the construction and function of STR neurons. In mice with Itga5 deficiency, PTEN overexpression lowered spine density and mushroom spines, but had no effect on spine density indicated in Figure 4.

We performed *in vivo* co-IP analysis using anti-Itga5 or anti-PTEN antibodies on protein lysates from striatal tissues to determine, if Itga5 interacts directly with PTEN. The PTEN antibody lowered Itga5, and vice versa display in Figure 4C. By co-transfecting Itga5 HA-tagged domain mutations and Flag-PTEN plasmids, we used immunoprecipitation to search for Itga5 or PTEN binding sites. The highly conserved GFFKR motif (Fig. 4D) 37, which helps inside-out signal transduction, was necessary for binding to PTEN protein, although Itga5's internal region and C-terminal motif were not (MEKAQLKPPATSDA). The connection requires the PTEN phosphatase domain, as found by using Itga5 to evaluate probable PTEN binding sites shown in Figure 4E. According to these results, the C-terminal motif of Itga5 interacts with the phosphatase domain of PTEN.

**Figure 5:**
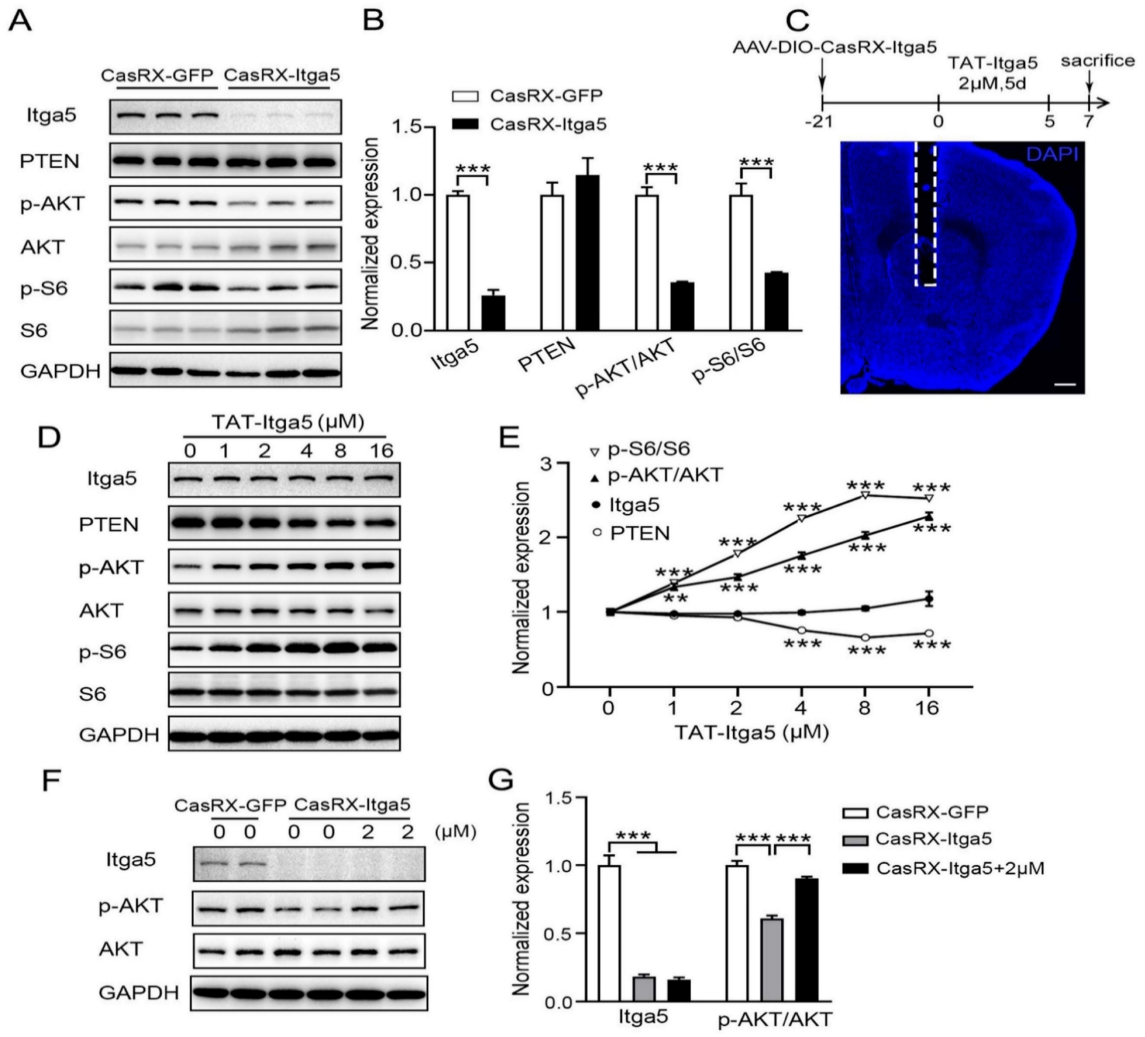
(A, B) Results showed a substantial drop in p-AKT and p-S6 protein levels, but no change in PTEN protein levels. (C) we perfused an Itga5 C-terminal binding peptide containing the cell-penetrating TAT sequence into the striatum of wild-type mice. (D,E) The levels of p-AKT and p-S6 proteins increased as TAT-Itga5 concentration rose.(F,G) When the TAT-Itga5 peptide was infused into the striatums of mice lacking Itga5, p-AKT protein levels were greatly restored.

According to several studies, the lipid phosphatase activity of PTEN suppresses the normal PI3K-AKT pathway 38, 39. The significance of Itga5 in PTEN signaling was then questioned. After suppressing Itga5, we detected a substantial drop in p-AKT and p-S6 protein levels, but no change in PTEN protein levels shows in Figure 5A-5B. To determine if Itga5 was directly responsible for this modification, we first synthesized an Itga5 C-terminal binding peptide containing the cell-penetrating TAT sequence (TAT-Itga5) 40, and perfused it into the striatum of wild-type mice shows in Figure 5C. The levels of p-AKT and p-S6 proteins increased as TAT-Itga5 concentration rose indicated in Figure 5D-5E. When the TAT-Itga5 peptide was infused into the striatums of mice lacking Itga5, p-AKT protein levels were greatly restored (Fig 5F-5G). TAT-Itga5 was unable to restore elevated AKT signaling, demonstrating that PTEN is necessary for Itga5 to control AKT signaling. These results suggest that Itga5 controls AKT signaling directly via its interaction with PTEN.

### TAT-Itga5 peptide rescues MPTP-induced motor impairments

**Figure 6:**
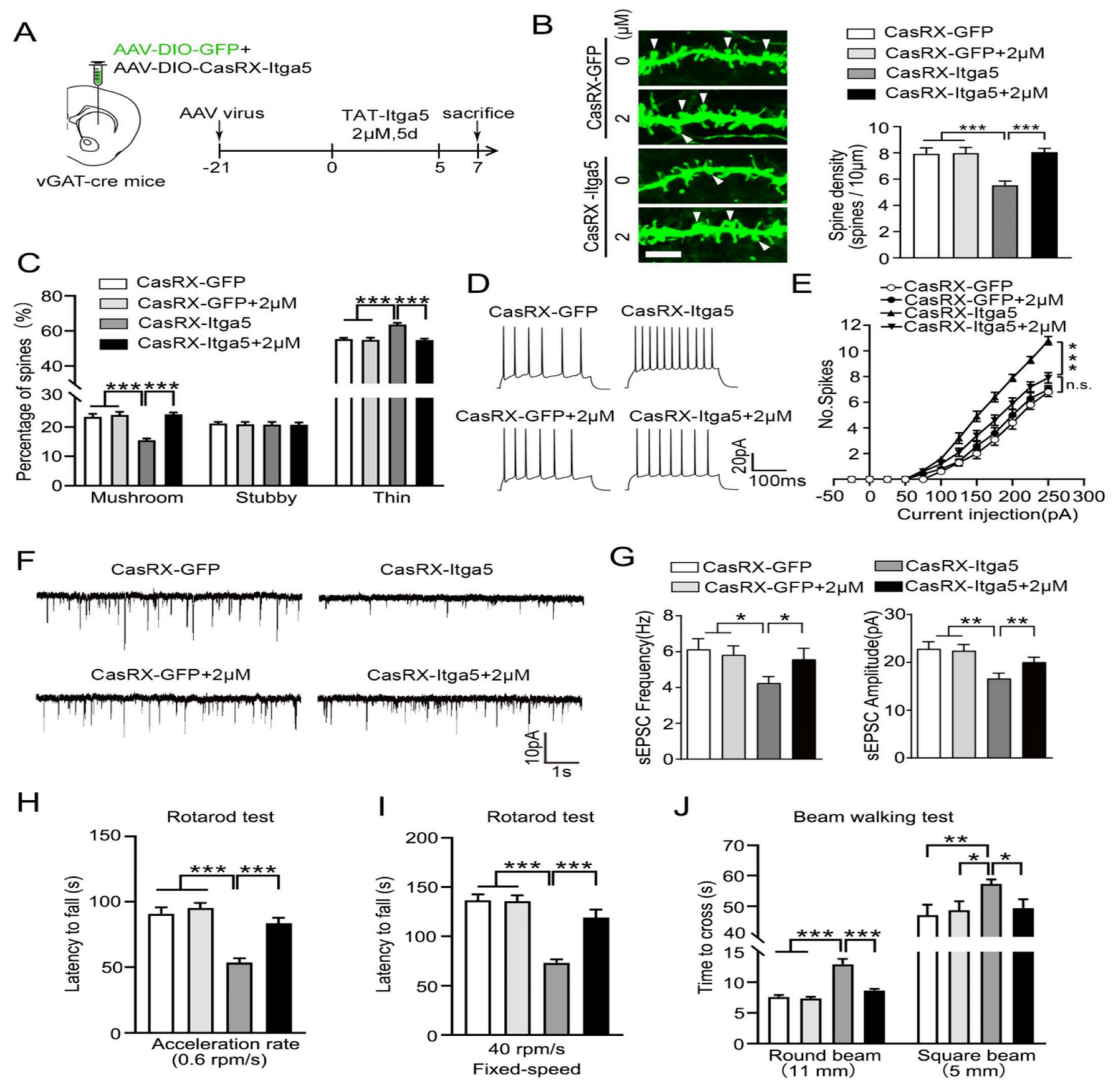
(A) Determine if PTEN signaling was the source of the Itga5-deficient animals' impairment. (B) In the striatum, the shape of MSN dendritic spines did not change between the GFP and GFP-2M groups. (C) CasRX-Itga5-2M displayed considerably increased spine density and proportion of mushroom spines than CasRX-Itga5. (D) In GFP control animals, perfusion with 2M TAT-Itga5 peptide showed no influence on the AP, sEPSC frequency, or amplitude of striatal MSNs. (E, F, G) AP was increased, sEPSC frequency was lowered, and amplitude was restored in CasRX-Itga5-2M mice. (H, I, J) CasRX-Itga5-2M animals regained motor skills equivalent to those of GFP control mice.

CasRx-GFP or CasRX-Itga5 expressing AAV was placed into the STR of vGAT-cre mice, followed by a three-week perfusion with 2M TAT-Itga5 peptide, to determine if PTEN signaling was the source of the Itga5-deficient animals' impairment shown in Figure 6A. In the striatum, the shape of MSN dendritic spines did not change between the GFP and GFP-2M groups. CasRX-Itga5-2M displayed considerably increased spine density and proportion of mushroom spines than CasRX-Itga5 indicates in Figure 6B-6C. In GFP control animals, perfusion with 2M TAT-Itga5 peptide showed no influence on the AP, sEPSC frequency, or amplitude of striatal MSNs. AP was increased, sEPSC frequency was lowered, and amplitude was restored in CasRX-Itga5-2M mice shows in Figure 6D-6G. CasRX-Itga5-2M animals regained motor skills equivalent to those of GFP control mice, allowing for enhanced synaptic development and function shows in Figure 6H-6J.

**Figure 7:**
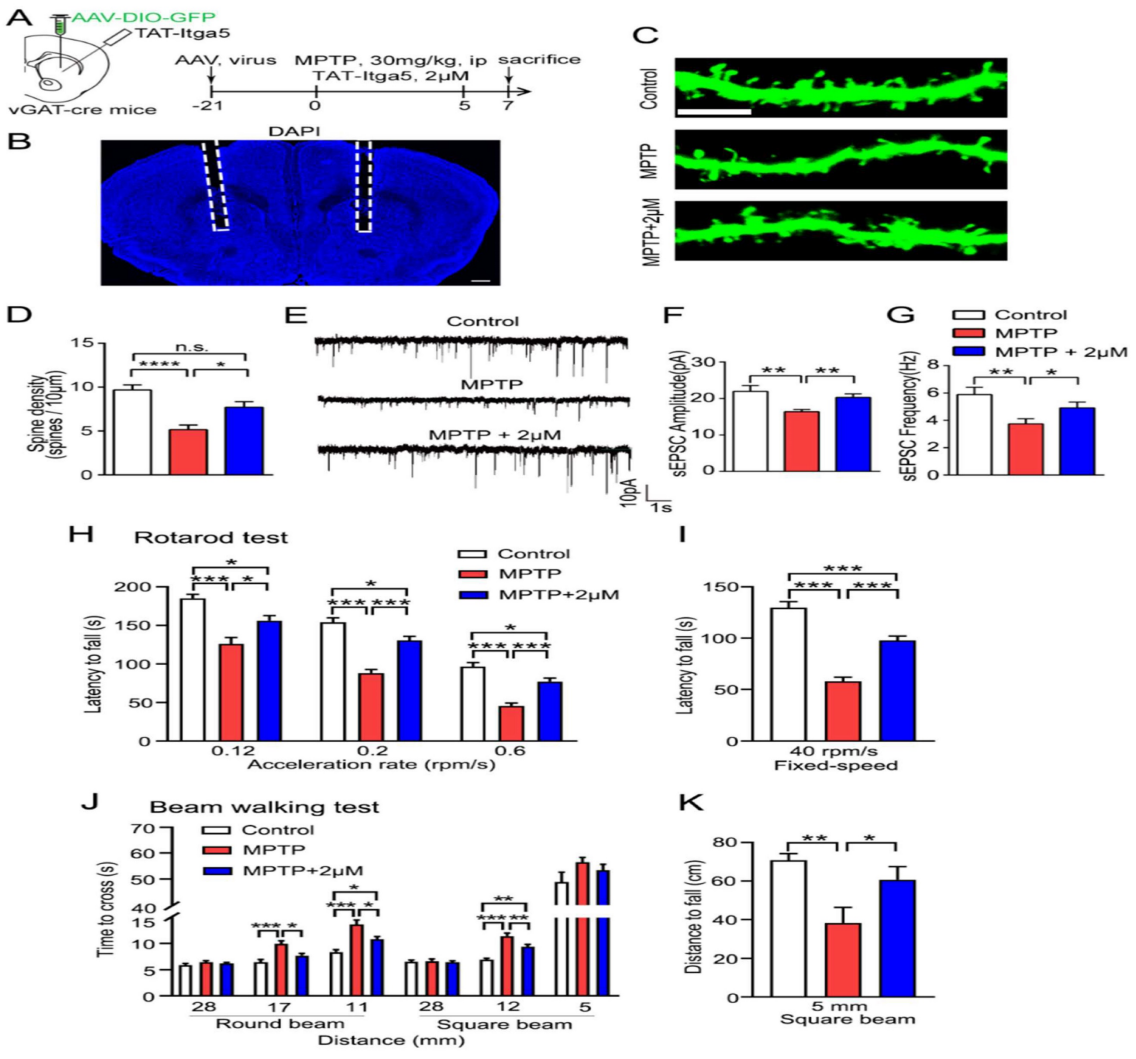
(A, B) TAT-Itga5 peptide might improve synaptic and motor performance in an MPTP-induced rat model of PD DIO-GFP-expressing AAV was delivered into the STR three weeks after an intraperitoneal (ip) injection of MPTP and perfusion of 2M TAT-Itga5 peptide to identify the STR GABA neurons in vGAT-cre rats. (C, D, E, F, G) The injection of 2M TAT-Itga5 peptide into MPTP rats restored spine density, sEPSC frequency and amplitude, but not the number of APs. (H, I, J, K). Moreover, 2M TAT-Itga5 peptide substantially slowed the progression of motor symptoms in mice with MPTP-induced encephalopathy in rotarod and balancing beam tests.

Finally, we evaluated whether the TAT-Itga5 peptide might improve synaptic and motor performance in an MPTP-induced rat model of PD DIO-GFP-expressing. AAV was delivered into the STR three weeks after an intraperitoneal (ip) injection of MPTP and perfusion of 2M TAT-Itga5 peptide to identify the STR GABA neurons in vGAT-cre rats shows in Figure 7A-7B. The injection of 2M TAT-Itga5 peptide into MPTP rats restored spine density, sEPSC frequency and amplitude, but not the number of APs indicated in Figure 7C-G. Moreover, 2M TAT-Itga5 peptide substantially slowed the progression of motor symptoms in mice with MPTP-induced encephalopathy in rotarod and balancing beam tests indicated in Figure 7H-7I. Our findings indicate that Itga5-PTEN signaling in striatal GABA neurons enhances synaptic transmission for motor coordination and may be a potential PD treatment target (Figure 7J-7K).

## Discussions

PD is distinguished by the loss of substantia nigra pars compacta cells and the subsequent denervation of nigrostriatal dopamine (DA) neurons (PD). Loss of DA regulation causes severe functional abnormalities in the striatum, which play a vital role in dyskinesia and cognitive disorders 41, 42. In a variety of animal models of PD (PD), striatal projection neurons (SPNs) demonstrate severe structural abnormalities in their dendritic spine density, shape, and ultrastructure 43, 44. Nevertheless, the molecular processes driving synaptic alterations in MPNs linked with PD remain unexplained. The expression of integrin 5 (Itga5) is dramatically decreased in mice with MPTP-induced Parkinsonism, according to our findings. Reduced endogenous Itga5 expression decreased the amount of Mushroom dendritic spines and enhanced the firing rate of SPNs. The reduction of PTEN activity has the potential to reverse spinal degeneration. Treatments with an Itga5 peptide that suppresses PTEN activity fix synaptic defects in SPNs and restore motor function in mice with MPTP-induced PD.

Rat and monkey models of PD, as well as postmortem striatal tissue from PD patients, have shown varying degrees of spine correction and plastic alterations in striatal SPNs 45. In addition, increased DA projection degeneration is associated by a substantial loss of dendritic spines 43, 46. However, the molecular mechanism controlling spine form in PD SPNs remains unknown. Expression of Itga5 in the striatum is significantly reduced by MPTP. Integrin represents a well-known group of cell surface adhesion receptors, and striatal neurons express Itga5 47. Integrin's play a crucial function in modifying the function of neurons to alter synaptic intensity in answer to activities 51 and are involved in a range of cellular processes 48-50. Itgb1, Itga3, Itga5, and Itga8 integrin's modulate synaptic formation and preserve LTP 52-54. Itga5 stimulates spine development and synapse formation in hippocampus neurons in culture 55. Since Itga5 is specifically expressed in the striatum, it is probable that it controls spine development shown in Figure S1. Itga5 expression in the striatum was inhibited using CRISPR-CaRX, resulting in a decrease in the number of Mushroom dendritic spines on SPNs.

Prior research on the architecture of SPNs in animals and humans with parkinsonian motor symptoms is vital, but insufficient for comprehending the connection between striatal spine loss and parkinsonian motor symptoms. This research seeks to establish if dendritic pathology is a comorbidity or a cause of parkinsonian motor symptoms. We noticed that suppressing Itga5 decreases SPN dendritic mushroom spines, which produces motor symptoms of PD Importantly, blocking Itga5 reduces motor symptoms of PD while preserving striatal dopamine release. As revealed in monkeys treated with MPTP, the degree of dendritic spine damage on striatal SPNs corresponds with the level of DA denervation, not with the severity degree of motor symptoms of PD. Our findings differ from those of earlier research due to alterations in study designs, dendritic pruning patterns, and behavioral markers. In other words, our findings show that the decreased dendritic spines of SPNs produce motor symptoms associated with PD, as opposed to being a consequence of the model of PD.

Integrin-signaling proteins such as focal adhesion kinase, src-family kinases, and integrin-linked kinase in migration, survival, differentiation, and motility play important roles 57-59. However, the intracellular signaling of integrins in the control of dendritic spines is not fully understood. PTEN modifies spine density and shape based on neuronal identification and categorization of spines 60. *In vivo*, PTEN knockdown increases spine density and frequency of excitatory postsynaptic current in the dentate gyrus. Pten deletion has no effect on the quantity of dendritic spines in basolateral amygdala neurons, but it does modify the morphology of spines from thin to mushroom-shaped 61. We demonstrate for the first time that *in vivo* PTEN affects the number and shape of SPN spines. PTEN signaling affects mEPSCs and action potentials in SPNs, as demonstrated by electrophysiological tests in which PTEN expression was increased or decreased through CRISPR-CasRx knockdown.

Injections of L-3, 4-dihydroxyphenylalanine (L-DOPA) are the most frequent therapy for PD (PD), and they initially reverse parkinsonian movement symptoms. In contrast, chronic L-DOPA administration causes dyskinesia or aberrant involuntary movements 62, 63. D1- and D2-MPNs have unique effects on L-DOPA-induced dyskinesia, which has characteristic of aberrant spine shape, impaired synaptic cell signaling, and changed EPSP-spike coupling 43, 64.

## Conclusion

Overall, our finding demonstrates the crucial role of the Itga5-PTEN-AKT pathway in synaptic and motor dysfunction in PD model. Chronic administration of TAT-Itga5 to the striatum of mice reverses MPTP-induced changes in SPN spine density, EPSP-spike and action potential. The treatment of mice with TAT-Itga5 markedly ameliorates the motor symptoms of MPTP-induced Parkinsonism. The Itga5-PTEN-AKT pathway is implicated in synaptic and motor dysfunction in PD, according to our research. The injection of TAT-Itga5 to the striatum of mice can relieve the motor function symptoms of PD as treatment for PD remains unchanged, our research would yield a better knowledge of how spine loss in STR neurons leads to motor deficits in the PD model, as well as a novel pathogenic target and potential solution for PD patients.

## Supplementary Material

Supplementary figure.

## Figures and Tables

**Table 1:** Primers for Real-time PCR

Gene	Sense	Anti-sense	Product size (bp)	
Itga2	TTGCTGTTGGCTATGGTTGC	TCTGTGGTCTCGTCCGTCTC		101
Itga5	CTCTGAAGATGCCCTACCAGATCCTGCC	TGATGATCCACAACGGGACACCATTGC		120
Itga6	ACCAGACTCTCAACTGCAGCGTCAA	CCTGGAACGAAGAACGAGAGAGGC		98
Itgam	GCCTCCCACAGCCAGCGGAT	GCCAGGTCCATCAAGCCATCCA		113
Itgav	AACAGCTTCAGCCTCATGAGAATGG	CACTGTTCACGAGAGATGTTGAAATCC		129
Itgb1	TTGCTGGAATTGTTCTTATTGGCCTTG	GTCCCACTTGGCATTCATTTTCTCCTT		119
Itgb2	CGTGGTAGGTGTCGTACTGATTGGTG	CGTGGTAGGTGTCGTACTGATTGGTG		106
Itgb4	ACCCGGCATGTGACCCAGGA	GGCGCTAGGAGAGGAGGCAAGG		147
Itgb5	TTGGCAGCATCCTCCTGATTGG	ATTTCATAACGGGCCCTGGAGC		121
Itgb8	TGCCTTCACCCTCACAATCTGTCTC	CGCAGATAGCTTGGGCCAGATAAA		122
Pcdh9	GACGAATTCTACGACCAGGCCTCTC	CAGCTAAACCTCGGGGACCCAG		100
Lgals1	GCATCACCTTTGACCAGGCTGAC	CTCCATCCGCCGCCATGTAGT		111
Lgasl3	GTAACACGAAGCAGGACAATAACTGGG	CCGCAACCTTGAAGTGGTCAGC		120
Lgals9	CCCCGTTTCAATGAGAATGCTGTT	AGCTCTGGCCTCGACTGAAGGG		109
Sema4c	TTCCCAAGAACATCACCGTTGTGTC	CAGGTTGTTCTGCAGGCAGGTCC		120
Sema4d	GGACATGAGCGGTGACACATCCT	GAACTTCCATACCACCCGGGCTA		136
Sema6d	ACATGAATGTCCTCATCACCTGCGT	CTGGGCGGATTCTGCGTCTTTAT		137
Sema7a	GACCTGCCCCATGGAGTCCC	AGGTTCTCAATGAACAGGATGCAGC		117
Fn1	TGATCCCCATGAAGCAACGTGCTAT	GCAGTTGTCACAGCGCCAGCC		136
Ecm1	GCGAGCTTCCATATCCAGAACAGG	CAGCAGAGGGCAGGGTCTTTCC		112
GAPDH	CTGAGCAAGAGAGGCCCTATCCC	GGTATTCAAGAGAGTAGGGAGGGCTC		124
				

## Abbreviations

PD: Parkinson's disease
SN: substantia nigra
IP: intraperitoneally
cDNA: supplementary DNA
AP: action potentials
STR: striatum
sEPSC: spontaneous excitatory postsynaptic currents
Itga5: integrin 5
L-DOPA: L-3, 4-dihydroxyphenylalanine
