# Supplementary material for: Itga5-PTEN signaling regulates striatal synaptic strength and motor coordination in Parkinson's disease
Source: Int J Biol Sci. 2024 Jun 11;20(9):3302–16. doi: 10.7150/ijbs.96116 (PMC11234218; doi:10.7150/ijbs.96116)

Figure S1

Supplemental Figure 1. Impaired neural excitability and synaptic transmission of STR GABA neurons in MPTP-induced PD model mice.

(a) Representative traces of the action potentials recorded from GFP labeled neurons in + 300 pA current injection for each experimental group. (b) Statistics showed that as the injection current increased (from -50 pA to + 300 pA), the number of spikes was significantly increased over the number seen in MPTP-induced PD mice (n = 15 neurons) compared to saline-administered controls (n = 18 neurons). (c) Representative sEPSCs traces recorded in the STR GABA neurons in saline-administered controls and MPTP-induced PD mice. (d) Quantification of basal frequency and amplitude of sEPSCs in GABA neurons from saline-administered controls and MPTP-induced PD mice (n = 15 neurons for each group). Statistical significance was determined by two-way ANOVA followed by Dunnett’s test (b) and unpaired Student's t test (d and e). Data are presented as the mean ± SEM. **p < 0.01; ***p < 0.001.

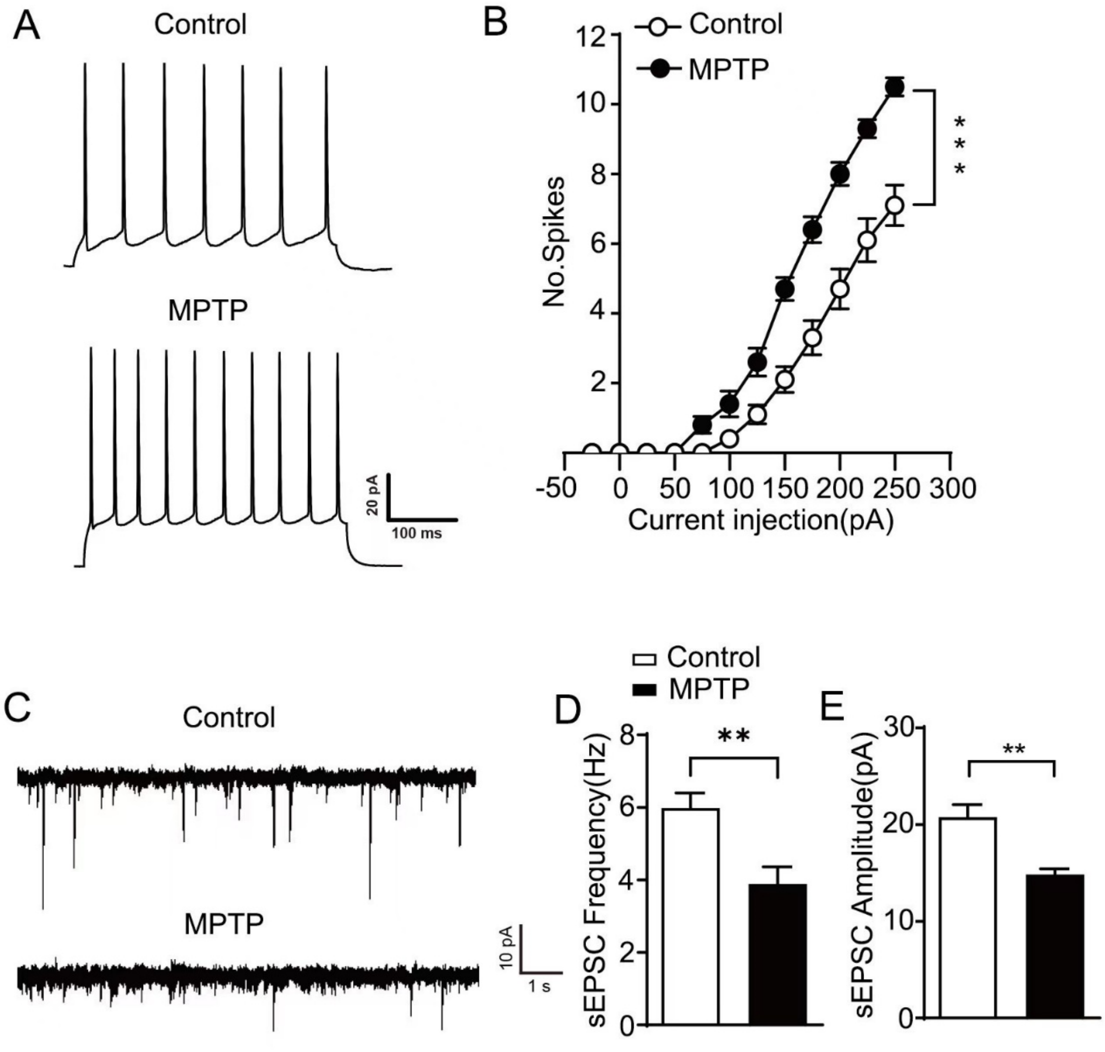

Supplement: Supplementary file 1 — Supplementary figure. [file ijbsv20p3302s1.pdf]
